# Treatment with soluble activin type IIB-receptor improves bone mass and strength in a mouse model of Duchenne muscular dystrophy

**DOI:** 10.1186/s12891-016-1366-3

**Published:** 2017-01-19

**Authors:** Tero Puolakkainen, Hongqian Ma, Heikki Kainulainen, Arja Pasternack, Timo Rantalainen, Olli Ritvos, Kristiina Heikinheimo, Juha J. Hulmi, Riku Kiviranta

**Affiliations:** 1 Department of Medical Biochemistry and Genetics, University of Turku, Kiinamyllynkatu 10, FI-20520 Turku, Finland; 2 Department of Endocrinology, Turku University Hospital, Turku, Finland; 3 Department of Biology of Physical Activity, University of Jyväskylä, Jyväskylä, Finland; 4 Department of Physiology, University of Helsinki, Helsinki, Finland; 5 Department of Oral and Maxillofacial Surgery, Institute of Dentistry, University of Turku, Turku, Finland; 6 Department of Oral Diagnostic Sciences, Institute of Dentistry, University of Eastern Finland, Kuopio, Finland; 7 Kuopio University Hospital, Kuopio, Finland; 8 Centre for Physical Activity and Nutrition Research, Deakin University, Melbourne, Australia; 9 Department of Physiology, Faculty of Medicine, University of Helsinki, Helsinki, Finland; 10 Institute of Dentistry, Faculty of Medicine, University of Helsinki, Helsinki, Finland

**Keywords:** Bone μCT, Bone-muscle interactions, TGF-βs, Animal models, Exercise

## Abstract

**Background:**

Inhibition of activin/myostatin pathway has emerged as a novel approach to increase muscle mass and bone strength. Duchenne muscular dystrophy (DMD) is a neuromuscular disorder that leads to progressive muscle degeneration and also high incidence of fractures. The aim of our study was to test whether inhibition of activin receptor IIB ligands with or without exercise could improve bone strength in the mdx mouse model for DMD.

**Methods:**

Thirty-two mdx mice were divided to running and non-running groups and to receive either PBS control or soluble activin type IIB-receptor (ActRIIB-Fc) once weekly for 7 weeks.

**Results:**

Treatment of mdx mice with ActRIIB-Fc resulted in significantly increased body and muscle weights in both sedentary and exercising mice. Femoral μCT analysis showed increased bone volume and trabecular number (BV/TV +80%, Tb.N +70%, *P* < 0.05) in both ActRIIB-Fc treated groups. Running also resulted in increased bone volume and trabecular number in PBS-treated mice. However, there was no significant difference in trabecular bone structure or volumetric bone mineral density between the ActRIIB-Fc and ActRIIB-Fc-R indicating that running did not further improve bone structure in ActRIIB-Fc-treated mice. ActRIIB-Fc increased bone mass also in vertebrae (BV/TV +20%, Tb.N +30%, *P* < 0.05) but the effects were more modest. The number of osteoclasts was decreased in histological analysis and the expression of several osteoblast marker genes was increased in ActRIIB-Fc treated mice suggesting decreased bone resorption and increased bone formation in these mice. Increased bone mass in femurs translated into enhanced bone strength in biomechanical testing as the maximum force and stiffness were significantly elevated in ActRIIB-Fc-treated mice.

**Conclusions:**

Our results indicate that treatment of mdx mice with the soluble ActRIIB-Fc results in a robust increase in bone mass, without any additive effect by voluntary running. Thus ActRIIB-Fc could be an attractive option in the treatment of musculoskeletal disorders.

## Background

Inhibition of the activin/myostatin pathway has recently emerged as a potential therapeutic approach for the treatment of osteoporosis. Activins are a group of multifunctional growth factors belonging to the TGF-β superfamily that play multiple roles in many physiological and systemic processes such as secretion of follistatin-stimulating hormone, wound healing, morphogenesis, and tooth formation [1–3]. Myostatin in turn inhibits muscle development and its inhibition leads to increased muscle mass [2, 3]. Activins and myostatin signal via activin type IIA or type IIB receptors [4]. The role of activins in bone physiology remained unclear until recent studies indicated that inhibition of activin receptor ligands leads to increased bone mass [5, 6]. In these experiments, soluble activin receptor-Fc fusion proteins were used as decoy receptors harvesting and thus inhibiting their ligands including activin A and myostatin. These studies suggested that inhibition of activin pathway could be a promising therapeutic target for metabolic bone diseases [7]. The use of a soluble activin type IIA-receptor has been shown to increase bone mass in several in vivo [5, 6, 8] models. The effects of soluble activin type IIB-receptor (ActRIIB-Fc) on bone metabolism have also been studied recently [9–12]. Furthermore, exercise, in addition to its various other health benefits, may have positive effects on bones of young individuals [13, 14]. Previously, the effects of exercise have also been investigated in a model of increased body mass through increased fat, not muscle mass [15]. In that model aerobic exercise does not further increase bone strength when compared to increased body mass alone suggesting interaction between physical activity and increased body mass. However, increasing body weight through muscle mass in combination with exercise has not been investigated before.

Duchenne muscular dystrophy (DMD) is a neuromuscular disease that is caused by a single mutation in the dystrophin gene. Patients suffering from this disorder are left immobilized and eventually are often deceased around the age of 20 [16]. Duchenne muscular dystrophy is also known to lead to secondary osteoporosis and increased fracture risk, at least in part due to the reduced mobility of DMD patients [16, 17]. By improving both muscle and bone function and strength, patient mobility could be prolonged and quality of life increased. The effects of exercise on bones in DMD as well as the interaction of the physical activity with ActRIIB-Fc ligand blocking are not known.

Muscle and bone tissues interact in multiple ways and muscle tissue can directly induce bone formation locally via several different molecular pathways [18, 19]. Further, muscle and body masses per se can also indirectly effect bone [20]. There is also reciprocal signaling from bone to muscle [21]. Myostatin and activins are inhibitors of muscle growth and their inhibition could have therapeutic implications in frailty and other diseases with muscle wasting [4, 22]. Intriguingly, ActRIIB-Fc can block both activin A as well as myostatin [23], that could potentially be very beneficial in conditions, such as frailty and DMD, which involve both muscle and bone [9].

Based on the previous data, we hypothesized that inhibition of the activin/myostatin pathway could provide a novel dual-effect treatment approach for musculoskeletal conditions improving both the muscle and bone properties. We aimed to answer the following questions: 1) does inhibition of activin receptor ligands with the use of a soluble activin type IIB-receptor (ActRIIB-Fc) affect bone volume and quality in a muscle dystrophy (mdx) mouse model [24] parallel to changes in muscle mass and 2) is there an interaction between ActRIIB-Fc treatment and low-intensity aerobic exercise.

## Methods

### Animals

In this experiment 6- to 7 week-old muscle dystrophy (mdx) male mice from a C57Bl/10ScSnJ background were used (Jackson Laboratory, Bar Harbor, Maine, USA). The mice were housed in standard laboratory conditions (temperature 22°C, light from 8:00 AM to 8:00 PM) and had free access to tap water and food pellets (R36, 4% fat, 55.7% carbohydrate, 18.5% protein, 3 kcal/g, Labfor, Stockholm Sweden).

### Experimental design

Thirty-two male mice were evenly divided into four groups: 1) PBS control group, 2) ActRIIB-Fc group, 3) PBS running group (PBS-R) and 4) ActRIIB-Fc running group (ActRIIB-Fc-R). PBS or ActRIIB-Fc was injected intraperitoneally once a week with a 5mg/kg dose. The chosen exercise modality was voluntary wheel running. To allow the treatment to take effect, the running wheels were locked during the first injection day and the following day preventing mice from exercising. On the last two days, the mice did not have access to running wheels so that the possible acute exercise effects would not affect the outcome. During the experiments all conditions were standardized. Mice were euthanized by cervical dislocation at the end of the experiment, after which tissue samples were harvested. One specimen from the ActRIIB-Fc-R group was moved to the ActRIIB-Fc group due to no voluntary running activity.

### Production of soluble ActRIIB-Fc

The ActRIIB-Fc protein used in the present study is similar, but not identical to the one originally generated by Se-Jin Lee [25]. The in house production of this recombinant protein has been described earlier [26]. Shortly, the protein contains the ectodomain of human ActRIIB linked to IgG1-Fc and was expressed in Chinese hamster ovary (CHO) cells grown in a suspension culture.

### Voluntary wheel running

As studies have shown that voluntary wheel running may benefit mdx mice in terms of muscle properties [27] and can also have positive effects on murine bones [15], it was chosen as the exercise modality for this study. The mice had free access to custom-made running wheels and the total running distance was recorded 24 h daily.

### Muscle mass and body weight measurement

The mice were weighed once every seven days to measure their body weight. After euthanization of the animals, gastrocnemius and quadriceps femoris muscles were collected and weighed immediately after dissection.

### Micro-computed tomography (μCT) analysis

X-Ray Micro-computed tomography of the distal femur and second lumbar vertebrae were done with SkyScan 1070 μCT scanner (SkyScan, Kontich, Belgium) to assess the microarchitecture of trabecular bone. Femur and lumbar vertebrae samples were placed in to plastic tubes and sealed with paraffin to minimize sample mobility. Scanning parameters included a voxel resolution of 5.33 μm, X-ray potential of 70kVp, current of 200uA and an integration time of 3900ms. The object rotated in 0.45° steps throughout the scanning for a total revolution of 182.45°.

Reconstruction of the scanned images was done (Nrecon 1.4, Skyscan) with identical settings (misalignment < 3, ring artifacts reduction 11, beam hardening correction 95%, and intensity gap 0.017–0.13). The regions of interest were drawn blindly (CTan 1.4.4, SkyScan). The corresponding starting points for the trabecular analysis were 120 layers (2.4mm) proximally of the distal femoral growth plate and 150 layers (3mm) distally of the cranial growth plate of the 2^nd^ lumbar vertebrae. The regions of interest (ROIs) were extended 150 layers (3mm) with a new ROI drawn every 10 layers (0.2mm). For the cortical analysis the ROI was drawn around the cortical bone of the femoral diaphysis, 600 layers (12mm) proximally of the distal growth plate, ranging 100 layers (2mm) in the proximal-distal plane. Finally the 3D, 2D and bone mineral density results were converted into numerical data for statistical analyses.

### Histological analysis

Femur samples were formalin-fixed, decalcified in EDTA, embedded in paraffin and sectioned using a microtome. The sectioned slides were deparaffinized, rehydrated and stained by either Hematoxylin & eosin or a tartrate resistant acid phosphatase (TRACP) stain and then counterstained with Mayer’s hematoxylin according to the manufacturer’s instructions. The trabecular bone architecture and osteoclasts were then analyzed blindly using Osteomeasure-histomorphometry work station (Osteometrics, USA). The analyzed area was defined as 800μm x 1200μm starting from 400μm proximally to the distal growth plate excluding the cortical bone borders. The region of interest was analyzed from three slices and the mean of each parameter was used as the corresponding value.

### Quantitative real-time PCR

To analyze the molecular effects of ActRIIB-Fc on bone C57Bl/6 F mice were administered with either PBS or ActRIIB-Fc (5mg/kg) once a week for 8 weeks (*n* = 6-7 per group). RNA was extracted from the right femur by performing an osteotomy to the distal and proximal ends of the bone and briefly centrifuging it to remove the bone marrow. The RNA was isolated using RNeasy minikit (Qiagen, Germany). The cDNA was synthesized from 1μg of RNA with SensiFAST probekit (Bioline, UK) before performing quantitative real-time PCR using iQ SYBR Green Supermix (Bio-Rad laboratories, USA). The relative mRNA expression levels were then quantified using the 2-ΔΔCT method. β-actin was used as the internal control.

**Table:** 

Primer	Forward sequence	Reverse sequence
*β-actin*	CGTGGGCCGCCCTAGGCACCA	TTGGCCTTAGGGTTCAGGGGG
*Runx2*	GCCCAGGCGTATTTCAGA	TGCCTGGCTCTTCTTACTGAG
*Col1a1*	GAGCGGAGAGTACTGGATCG	GCTTCTTTTCCTTGGGGTTC
*Opn*	ATCTGGGTGCAGGCTGTAA	CCCGGTGAAAGTGACTGATT
*Dmp-1*	TTGGGATGCGATTCCTCTAC	GGTTTTGACCTTGTGGGAAA
*Sost*	GCAGCTGTACTCGGACACATC	TCCTGAGAACAACCAGACCA
*Dkk-1*	GACAACTACCAGCCCTACCC	GATCTGTACACCTCCGACGC
*Rankl*	TGAAGACACACTACCTGACTCCTG	CCACAATGTGTTGCAGTTCC
*Opg*	ACCCAGAAACTGGTCATCAGC	CTGCAATACACACACTCATCACT
*TrAcp5*	CGTCTCTGCACAGATTGCAT	AAGCGCAAACGGTAGTAAGG
*Ctsk*	AGGCATTGACTCTGAAGATGCT	TCCCCACAGGAATCTCTCTG

### Testing of mechanical properties

The mechanical properties of the femur and tibia were determined by a three-point bending test using biomechanical testing device (Mecmesin, West Sussex, UK). The femur was positioned horizontally with the anterior surface upward, centered on the supports (span = 9mm); and the middle point of the femoral and tibial shaft were vertically compressed at a constant speed of 4.5mm/min, and data was collected at a sampling rate of 10Hz until failure. The measured data was converted to a load-displacement curve in a monitoring recorder linked to the tester. The maximum force and deformation and the break force and deformation were read respectively from the highest point of the load-deformation curve and from the failure point. Stiffness was calculated as the slope of the curve, which was fitted in the linear part of the load-deformation curve.

### Statistical analyses

Two-way analysis of variance (2x2 ANOVA) was used for statistical evaluation and Student’s *T*-test as a post hoc test with the statistical significance set to *p* < 0.05.

## Results

### Treatment with soluble ActRIIB-Fc increases body weight and muscle mass

This is a follow-up study of Hulmi et al. 2013 and the muscle and body weight results have been published previously [28, 29]. In brief, our results show that the muscle mass increased in the ActRIIB-Fc treated groups. A significant change in the masses of both the gastrocnemius muscles and quadriceps femoris muscles was noted when comparing ActRIIB-Fc and PBS groups to each other. The running ActRIIB-Fc-R mice also developed larger muscle mass compared to the running PBS-R group but changes were more modest compared to the non-running groups.

As expected both active and sedentary ActRIIB-Fc treated mice also had a significant increase in body weight compared to their respective PBS controls. However, both running groups gained less weight during the experiment and weighed less also at the end compared to non-runners, mainly due to decreased fat mass as published earlier [15]. Therefore at the end of the experiment the bodyweight was equal in the ActRIIB-Fc-R and PBS groups. The final mean weights for the mice per group were as follows: PBS 30.3 ± 0.8g, ActRIIB-Fc 34.0 ± 1.9g, PBS-R 29.7 ± 1.7g and ActRIIB-Fc-R 31.6 ± 2.3g.

### ActRIIB-Fc increases bone volume and bone mineral density in appendicular skeleton

**Fig. 1:**
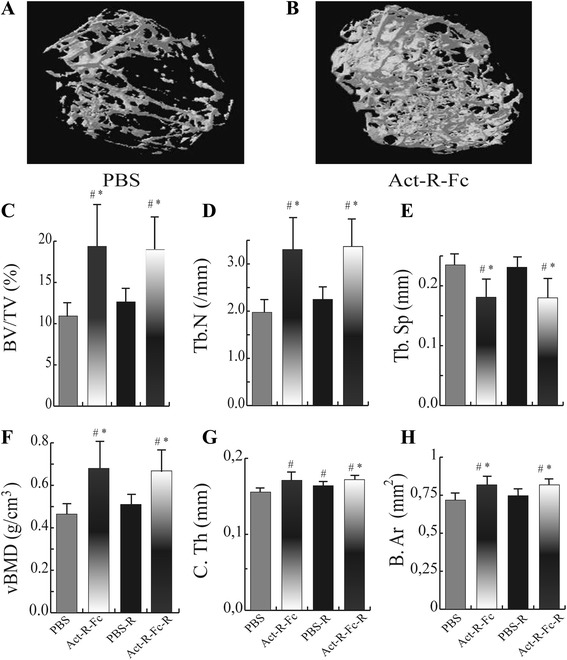
Reconstructed 3D images of the distal femur of control (**a**) and Act-R-Fc treated mice (**b**) showing a significant increase in bone mass in the treated group. μCT analysis of the distal femur showing (**c**) increased bone volume (BV/TV), (**d**) trabecular numbers (Tb.N), (**e**) decreased trabecular separation and (**f**) increased volumetric bone mineral density (vBMD) in ActRIIB-Fc-treated mice compared to controls. Significant increases in (**g**) cortical thickness and (**h**) mean total cross-area parameters were also noted**.** Running combined with ActRIIB-Fc treatment did not further improve these parameters *N* = 7-9 per group. # = *p* < 0.05 compared to PBS, * = *p* < 0.05 compared to PBS-R

The μCT analysis of the distal femur showed a remarkable increase in bone mass in the ActRIIB-Fc-treated mice (ActRIIB-Fc effect ANOVA: *p* < 0.001) and this effect of ActRIIB-Fc was seen in both running and sedentary mice (Fig. 1). Bone volume and trabecular number (Fig. 1c-d) were increased in ActRIIB-Fc treated sedentary mice by over 80% and over 70% (*p* < 0.001), respectively. Volumetric bone mineral density (vBMD) (Fig. 1f) increased in the ActRIIB-Fc treatment group and the separation (Fig. 1e) between trabeculae also decreased (ActRIIB-Fc effect *p* < 0.001). These results demonstrate larger, more numerous trabeculae that are located more closely to each other resulting in a more dense tissue compared to PBS controls, when activin receptor ligand signaling is inhibited. Running alone had little effect on bone volume and trabecular number in PBS-treated mice. In addition, there was no significant difference in trabecular bone structure or vBMD between the ActRIIB-Fc and ActRIIB-Fc-R groups indicating that running did not further improve bone architecture in ActRIIB-Fc-treated mice (ANOVA ActRIIB-Fc x running interaction effect *p* = 0.434). The cortical bone analysis of the femoral mid-shaft that ActRIIB-Fc treatment, with and without running, increased cortical thickness by 14% (*p* < 0.05 in both) when comparing to PBS controls (Fig. 1g). Running alone also resulted in a 10% increase (*p* < 0.05 compared to sedentary PBS group) in cortical thickness. Mean total cross-sectional bone area was also increased by 10% by ActRIIB-Fc treatment and running (Fig. 1h). However, there was no additional effect on either cortical thickness or area by the combination of ActRIIB-Fc and running (ANOVA ActRIIB-Fc x running interaction effect *p* = 0.436 and *p* = 0.421, respectively).

### ActRIIB-Fc treatment results in increased bone mass also in axial skeleton

We also wanted to assess whether the treatment with ActRIIB-Fc only affected weight bearing long bones or if the axial skeleton was involved as well. For this purpose we performed μCT analysis on the second lumbar vertebrae.

**Fig. 2:**
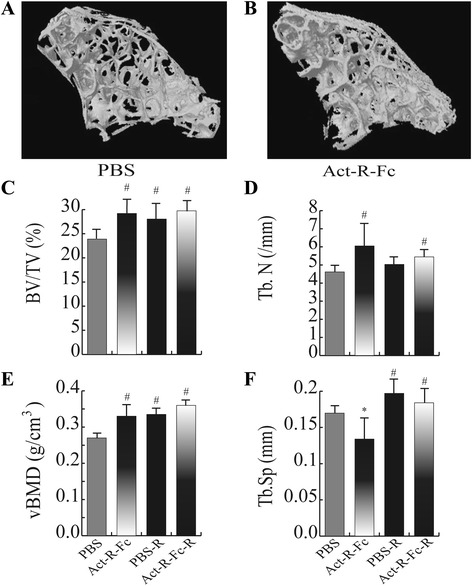
Reconstructed 3D images of the 2^nd^ lumbar vertebrae also showing a noticeable difference in bone mass between the control (**a**) and treatment group (**b**). Similar to the distal femur, (**c**) bone volume (BV/TV), (**d**) trabecular number (Tb.N) and (**e**) volumetric bone mineral density (vBMD) were increased while (**f**) trabecular separation (Tb. Sp) was decreased in ActRIIB-Fc-treated mice compared to controls. ActRIIB-Fc and running did not result in further beneficial effect. # = *p* < 0.05 compared to PBS, * = *p* < 0.05 compared to PBS-R

The ActRIIB-Fc treatment-induced changes in the vertebrae were similar but more modest compared to the distal femur (Fig. 2). Bone volume (Fig. 2c) was increased by 20% and trabecular number (Fig. 2d) by 30% in the ActRIIB-Fc-treated mice compared to PBS group (*p* < 0.001). Similar to the femur analysis, bone mineral density (Fig. 2e) was also increased and trabecular separation (Fig. 2f) decreased (ActRIIB-Fc effect *p* < 0.05). Running seemed to induce an increase in trabecular bone volume and vBMD in vertebrae when comparing PBS-R to the PBS-controls (running effect *p* < 0.05). As noted previously for femur, the combined effect of ActRIIB-Fc and running on vertebral bone did not significantly differ from the effect of ActRIIB-Fc treatment alone.

### ActRIIB-Fc decreases the number of osteoclasts on trabecular bone

**Fig. 3:**
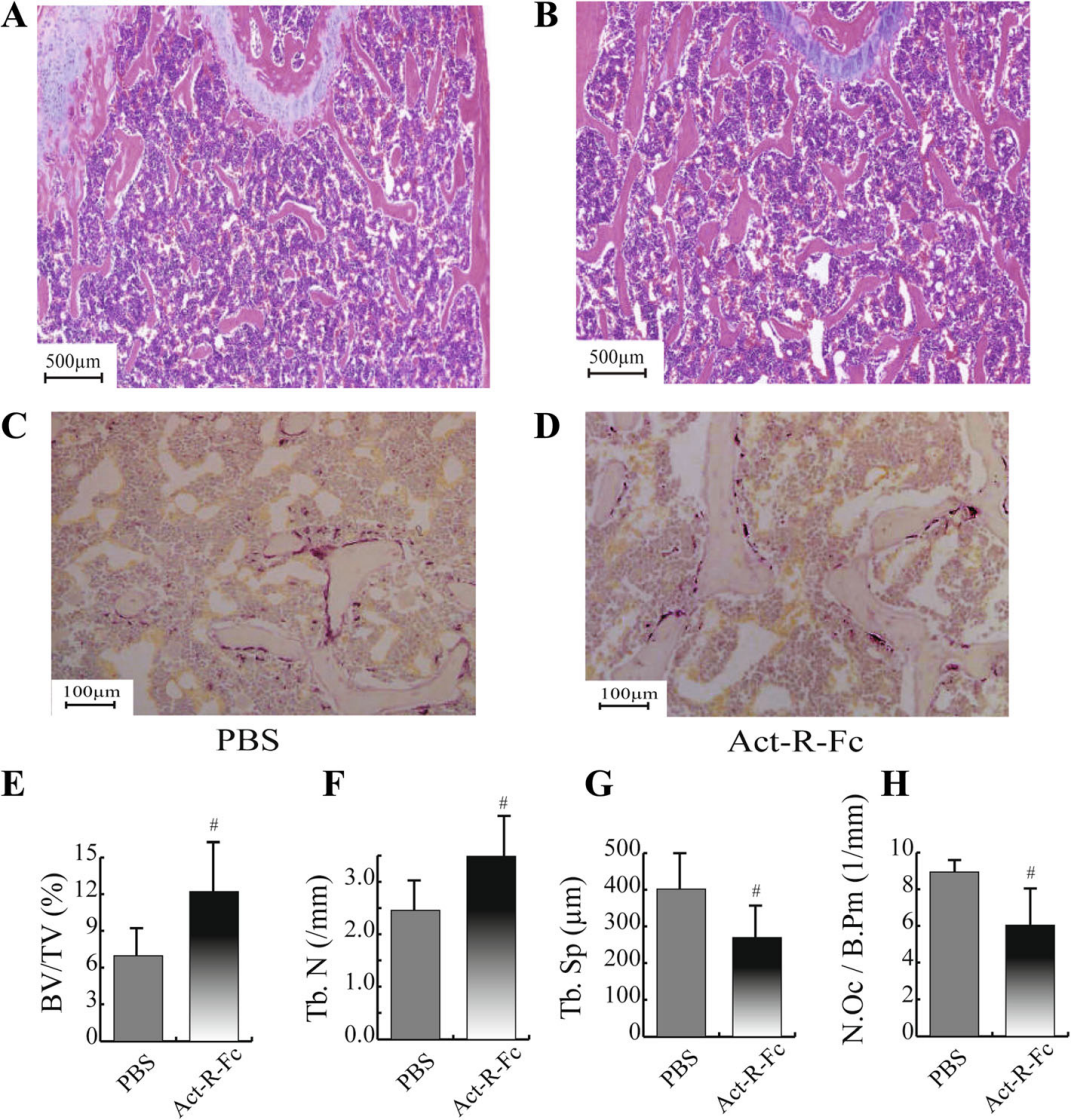
Histological images (H&E staining) of the distal femur showing an increase of trabecular numbers in ActRIIB-Fc treated group (**b**) compared to PBS controls (**a**). TRACP staining of distal femurs of control (**c**) and Act-R-Fc treated mice (**d**). Histomorphometric analysis showed increased bone volume (**e**) and trabecular number (**f**) and decreased trabecular separation (**g**) as in μCT. Interestingly, the number of TRACP positive osteoclasts was reduced in Act-R-Fc-treated mice (**h**). The region of interest from which the trabecular islands and the stained osteoclasts were analyzed consisted of an 800μm x 1200μm area near the distal growth plate. *N* = 8-9 per group. # = *p* < 0.05 compared to PBS

Histological analysis of distal femur confirmed our findings in μCT analyses of increased bone volume per tissue volume and trabecular number in the ActRIIB-Fc-treated mice (Fig. 3). Interestingly, a decrease in osteoclast number (N.Oc/B.Pm) was also observed (Fig. 3h). This suggests that in addition to its published effects on bone formation ActRIIB-Fc treatment suppresses osteoclast differentiation and therefore inhibits bone resorption.

### ActRIIB-Fc treatment increases expression of osteoblast markers and decreases expression of osteoclast markers

**Fig. 4:**
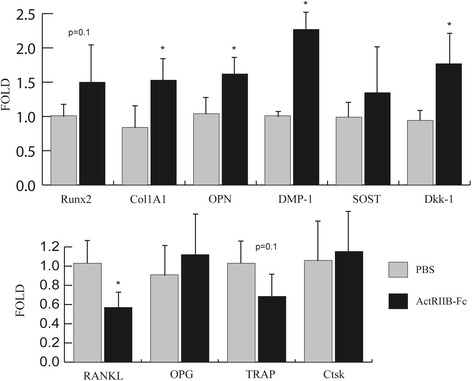
qPCR analyses show changes in the expression of key markers for bone metabolism. Abbreviations in order: Runt related transcription factor 2, Type I collagen, Osteopontin, Dentin matrix protein 1, Sclerostin, Dickkopf-related protein 1, Receptor activator of nuclear factor kappa-B ligand, Osteoprotegerin, Tartrate resistant acid phosphatase, Cathepsin K. *N* = 6-7 per group. * = p ≤ 0.05 compared to PBS group

To further investigate the mechanisms behind these effects we treated C57Bl mice with ActRIIB-Fc or vehicle, collected bone samples and measured the expression of essential osteoblast and osteoclast marker genes. qPCR analysis (Fig. 4) revealed increased expression of characteristic osteoblast markers (Col1A1 + 80% *p* = 0.05, OPN +55% *p* < 0.05) and osteocyte marker (DMP-1 + 125% *p* < 0.01) as well as decreased expression of RANKL (-44% *p* < 0.01) a regulator of bone resorption. There was also a trend of decreased TRAP expression (-33% *p* = 0.1) in the treated group. These results suggest that ActRIIB-Fc simultaneously increases osteoblast induced bone formation and decreases bone resorption.

### Treatment with ActRIIB-Fc improves mechanical strength

**Fig. 5:**
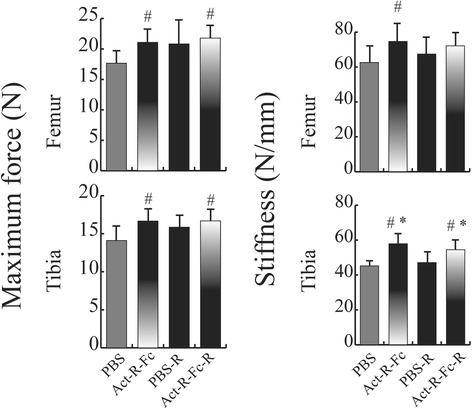
Biomechanical testing of femurs and tibias. A significant increase in both maximum force and stiffness was measured in the Act-R-Fc treated mice compared to the control group. However there was no statistical significance between the ActRIIB-Fc-treated and control running groups. *N* = 7-9 per group. # = *p* < 0.05 compared to PBS, * = *p* < 0.05 compared to PBS-R

To test whether treatment with ActRIIB-Fc would also improve the mechanical properties of long bones, we performed a three point bending test on femurs and tibias. There was a significant increase in stiffness and bone strength in the ActRIIB-Fc-treated compared to the PBS group in sedentary mice (Fig. 5). Running alone resulted in a trend of stiffer and stronger bones but this did not appear to be statistically significant. In addition, voluntary running did not further improve bone biomechanical properties in the running ActRIIB-Fc-treated mice.

To further explore the effects of ActRIIB-Fc treatment on bone tissue, the results were then adjusted with body mass acting as the covariate. After adjustment minor changes could only be seen in the μCT analysis of the vertebrae that did not affect the interpretation of the results. Therefore the differences between the body weights of the mice did not seem to significantly affect the results of our analyses. The datasets analyzed during the current study are available from the corresponding author upon request.

## Discussion

In this study we evaluated the effects of inhibition of ActRIIB ligands on bone and muscle tissue using a soluble activin type IIB-receptor in an mdx mouse model. We also aimed to test whether voluntary physical activity combined with ActRIIB-Fc treatment would have an effect on these tissues. Our results indeed confirm our hypotheses. First, we were able to show a significant increase in bone mass in ActRIIB-Fc treated mice compared to PBS treated control mice. Second, our findings demonstrate that ActRIIB-Fc affects both appendicular and axial bone mass and beneficially modifies biomechanical properties of long bones. Finally, although exercise alone had some positive effects on bone structure, combination of running exercise with ActRIIB-Fc did not further increase bone mass or strength compared to ActRIIB-Fc treatment alone.

Our μCT results of the distal femur showed that the ActRIIB-Fc treatment resulted in increased vBMD, number of bone trabeculae and increased bone volume. The separation of trabeculae was also decreased. Our findings are consistent with previous reports demonstrating that treatment with either ActRIIA-Fc [6, 8] or ActRIIB-Fc [9, 10] resulted in increased bone volume. Increased BMD in trabecular bone also suggests that at least part of the effect could be derived from decreased bone resorption, possibly due to slower bone turnover and prolonged secondary mineralization and filling of resorption spaces. However, it was interesting to note that the differences between ActRIIB-Fc and ActRIIB-Fc-R groups were miniscule showing that physical activity did not have a noticeable further effect on trabecular bone in the murine femur in this model. ActRIIB-Fc-R mice ran significantly less especially in the beginning of the study as previously reported [26], which could at least in part explain the lack of additional effect of running on bone mass or strength in the ActRIIB-Fc-R group. The non-significant change between PBS and PBS running group in bone mineral density was also surprising suggesting that physical activity in a form of voluntary running does not greatly affect bone quality in long bones in young mdx mice.

In humans, weight bearing exercise has positive effects on the bones of young individuals and voluntary running may have positive effects on murine bones [30]. However, as often rather high intensity/volume of exercise is needed for positive effects on bone, our training modality might have been of too low intensity to induce more robust effects on bone mass [14]. In contrast to our results, Hamrick et al. suggested that the combination of exercise and increased muscle mass in myostatin-deficient mice has a much greater effect on bone strength than exercise or muscle mass alone [31]. This could be explained by the fact that the myostatin-deficient mice used in their study had increased lean mass postnatally and this increased contractile forces induced by locomotion and resulted in a more powerful mechanotransduction effect on bones. In addition, their exercise regime based on force exercise was most likely more intense compared to our method of voluntary exercise. Finally, as Hamrick et al. analyzed the radius, they stated that their effects could be partly explained by the difference in the load induced by the curvature of the bone they analyzed.

The effect of ActRIIB-Fc treatment was also seen in second lumbar vertebrae but it was more modest than in distal femur analysis. Bone volume was increased by 22% in vertebrae compared to 83% in the femur. Our results on lumbar vertebrae are consistent with the article published by Bialek et al., in which they used normal C57Bl mice [9]. As in femur, voluntary physical activity combined with ActRIIB-Fc treatment did not have a synergistic effect in vertebrae. Trabecular numbers increased only marginally and the increases in bone volume and volumetric bone mineral density were not statistically significant.

Improved cortical geometry in long bones also translated into significantly improved biomechanical properties as both the maximal failure load as well as stiffness increased in the femurs and tibias of ActRIIB-Fc treated mice. Bialek et al [9] also reported that ActRIIB-Fc treatment increased bone strength when compared to vehicle group but found this only in the L4 vertebrae. In our study voluntary running did not have statistically significant effect on bone strength although there was a positive trend in both failure load and stiffness. This lack of effect could be due to the relatively small sample size.

Histological analysis of the distal femur comparing PBS and ActRIIB-Fc groups was done to assess the effect of blockage of ActRIIB-ligands on osteoclast parameters. Histological analysis confirmed the increase in bone volume and trabecular number as was noticed in μCT. However, we also found a significant decrease in the number of osteoclasts per bone perimeter in the ActRIIB-Fc treated animals. This suggests a suppression of osteoclast differentiation and subsequently bone resorption that has not previously been reported with ActRIIB-Fc. As discussed above, this finding is in agreement with the increased trabecular vBMD in the distal femur observed with μCT. Activin A has been shown to induce osteoclast differentiation in vitro and in vivo [32–35], although some reports suggest a negative effect on survival and motility of mature osteoclasts [36]. The decreased osteoclast number in our study supports the role for activin A to induce osteoclast differentiation, although we cannot exclude the possible effect of other ligands binding to ActRIIB-Fc. In addition, ActRIIB ligand inhibition could also affect the osteoblast-dependent regulation of osteoclastogenesis. Previous studies have shown that blocking of activin receptor ligands results in increased bone formation translating into increased bone mass. Unfortunately, the simultaneous signaling and metabolic analyses of muscle tissues performed in this study [26] and the limited number of animals available prevented us from using fluorochromes to measure bone formation in mice. However, based on the very robust increase in trabecular bone volume, gene expression discussed below and the previously published data, it is very likely that ActRIIB-Fc molecule used in our study also induced an increase in bone formation.

Our results also provide novel data regarding the molecular mechanism behind the effects of ActRIIB-Fc on bone growth. We were able to show that ActRIIB-Fc induces an increase in osteoblast and osteocyte gene markers suggesting that ActRIIB-Fc indeed also has an anabolic effect on bone. Furthermore we were able to confirm our hypothesis of ActRIIB-Fc acting as a suppressor of osteoclast activity as the expression of osteoclast markers decreased noticeably. Interestingly, expression of DKK-1 and sclerostin, key markers for negative regulation of WNT signaling, also significantly increased. This could be due to negative feedback loop induced by increased bone mass and/or reflect enhanced Wnt signaling. Alternatively, the concomitantly increased osteocyte number could also contribute to the increased expression of DKK1 and sclerostin.

Based on the present experiment, the molecular basis for the effects of ActRIIB-Fc treatment and running on bone tissue, independent of increased body and muscle mass, remain unclear. If ActRIIB-Fc and running have independent signaling pathways for bone adaption, one would have expected an additive effect. However, investigating this interaction is encumbered by the marked effect of running on body mass, and the inextricable link between the body and skeletal size. On the other hand, adjusting the data to body weight did not significantly alter the results on bone structure or strength. Considering the effectiveness of the interventions in isolation, the results indicated a clear and robust skeletal effect by ActRIIB-Fc. However, judging by the PBS groups the effects of the running intervention on skeletal mass were much more modest, and could not be observed in the femur analysis. Clearly, from the two interventions applied in the present study, ActRIIB-Fc was more effective, as we expected. Therefore our study provides promising evidence that ActRIIB-Fc could be applied as a therapeutic agent in musculoskeletal disorders where physical activity is limited.

It is also notable that as soluble activin receptors also bind other growth factors in addition to activin A [23, 37]. It remains unclear which is/are the main effectors inducing the observed alterations. Activin A suppresses bone formation and induces resorption and is therefore the primary suspect for direct bone effects of ActRIIB-Fc, but inhibition of other growth factors may also contribute. Most likely the majority of the effect on bone is derived from direct regulation of bone cell functions. However, due to the known muscle-bone interactions it is also possible that some of the positive effects on bone could stem from simultaneous increase in muscle mass either via increased body mass or by direct molecular cross talk between muscle and bone tissues. As adjusting for the body mass did not alter the results the effect would likely be direct signaling between the tissues. Further molecular studies are needed to clarify this question.

## Conclusions

In conclusion, we show here that inhibition of activin receptor ligand signaling using ActRIIB-Fc positively affects bone mass and quality and subsequently bone mechanical strength, without further positive effect by physical exercise. Soluble ActRIIB-Fc could provide an intriguing approach for the treatment of coexisting muscle and bone loss in many diseases, aging and in injuries.

## Abbreviations

ActRIIB-Fc: Activin type IIB receptor fusion protein
BV/TV: Bone volume per tissue volume
Col1a1: Collagen type I
DMD: Duchenne muscular dystrophy
Mdx: Dystrophic mouse with point mutation in the dystrophin gene
N.Oc/B.Pm: Number of osteoclasts per bone perimeter
OPN: Osteopontin
PBS: Phosphate buffer solution
RANKL: Receptor activator of nuclear factor kappa-B ligand
Tb.N: Trabecular number
TGF-β: Transforming growth factor β
Trap: Tartrate-resistant acid phosphatase
vBMD: Volumetric bone mineral density
μCT: Micro-computed tomography
